# Supplementary material for: Toxin Homology Domain in Plant Type 2 Prolyl 4-Hydroxylases Acts as a Golgi Localization Domain
Source: Cells. 2024 Jul 9;13(14):1170. doi: 10.3390/cells13141170 (PMC11275109; doi:10.3390/cells13141170)
Supplement: Supplementary file 1 [file cells-13-01170-s001.zip › Moriguch and Matsuoka, legends to suppl figures.pdf]

Legends to Supplemental figures: Moriguchi and Matsuoka, Toxin-Homology Domain of Plant Type 2 Prolyl 4-Hydroxylases act as Golgi Targeting Domain.

Figure S1. Amino acids sequences of NtP4H2.1, NtP4H2.2 and related proteins. (A)Comparison of the amino acid sequences of type 2 P4Hs from tobacco (NtP4H2.1 and NtP4H2.2), Arabidopsis (At-PH-2 and AT3G28480) and rice (AK059750 and AK103739) and type 1 P4Hs from tobacco (NtP4H1.1) and Arabidopsis (At-PH-1). The conserved HxD motif and a histidine residue, which are important for activity, are indicated by asterisks. The tox1 domains in type 2 P4Hs are boxed. The six conserved cysteine residues in the tox1 domain are indicated. Predicted N-terminal signal peptides in NtP4H2.1 and NtP4H2.2 are underlined. Residues conserved in more than 5 sequences are colored in gray. (B) Comparison of the amino acid sequences of tox1 domains from plant type 2 P4Hs, BgK and ShK. The six conserved cysteine residues are indicated. Residues conserved in more than 3 sequences are colored in gray. (C) Characterization of the NtP4H2.2 antibody. Total proteins were extracted from 5-day old tobacco BY-2 cells, resolved by SDS-PAGE and blotted onto a PVDF membrane. The membranes were probed with pre-immune serum (pre-immune) or affinity-purified NtP4H2.2 antibody from immune serum (immune). The arrow indicates the size of NtP4H2.2 (33.9 kDa) without a signal peptide. The asterisk indicates non-specific bands. (D) Schematic representations of the fusion constructs used in this study. Numbers indicates the amino acid position of each P4Hs used for the fusion constructs. The positions of the tox1 domain in NtP4H2.1 and NtP4H2.2 are indicated. The amino acid residues of the tox1 domain used for preparing the GFP-tox1 constructs are shown. The gray box indicates the linker residues (Gly-Gly-Thr-Thr) between GFP and the inserted protein.

Figure S2. Localization of GFP fused with tox1 domain from type 2 P4Hs in BY-2 cells.
(A) and (B), GFP-tox1(NtP4H2.1)(A) and GFP-tox1(NtP4H2.2)(B) were expressed in BY-2 cells expressing NtP4H1.1-mRFP. GFP and RFP fluorescences were analyzed by confocal microscopy. (C), to (F), tox1 domain from rice AK059759 (C) and AK103739 (D), or Arabidopsis At-PH-2(E) and AT3G028480 (F) were fused with spo41(I28G,P36Q)-GFP and expressed in BY-2 cells expressing NtP4H1.1-mRFP. GFP and RFP fluorescences were analyzed by confocal microscopy. Scale bar=10μm.

Figure S3. The effects of brefeldin A (BFA) treatments on the localization of GFP fused with tobacco type 2 P4Hs or their tox1 domain. (A) The distribution of ER in tobacco BY-2 cells visualized by the expression of KDEL-tagged GFP. (B) Effects of BFA on spo41(I28G,P36Q)- GFP localization in BY-2 cells. (C-H) Effects of BFA on GFP -NtP4H2.1(C), GFP -tox1(NtP4H2.1) (D), GFP-NtP4H2.2(E), GFP-tox1(NtP4H2.2) (F), GFP-NtP4H2.1(Δtox1) (G), and GFP-NtP4H2.2(Δtox1) (H) localization in BY-2 cells expressing NtP4H1.1-mRFP. Cells were treated with 14.8 μM BFA for 120 min. After the BFA treatment, GFP and RFP fluorescences were analyzed by confocal microscopy. DMSO treatments represent control treatments. Scale bar=10μm.
