# Supplementary figures and images for: Toxin Homology Domain in Plant Type 2 Prolyl 4-Hydroxylases Acts as a Golgi Localization Domain
Source: Cells. 2024 Jul 9;13(14):1170. doi: 10.3390/cells13141170 (PMC11275109; doi:10.3390/cells13141170)

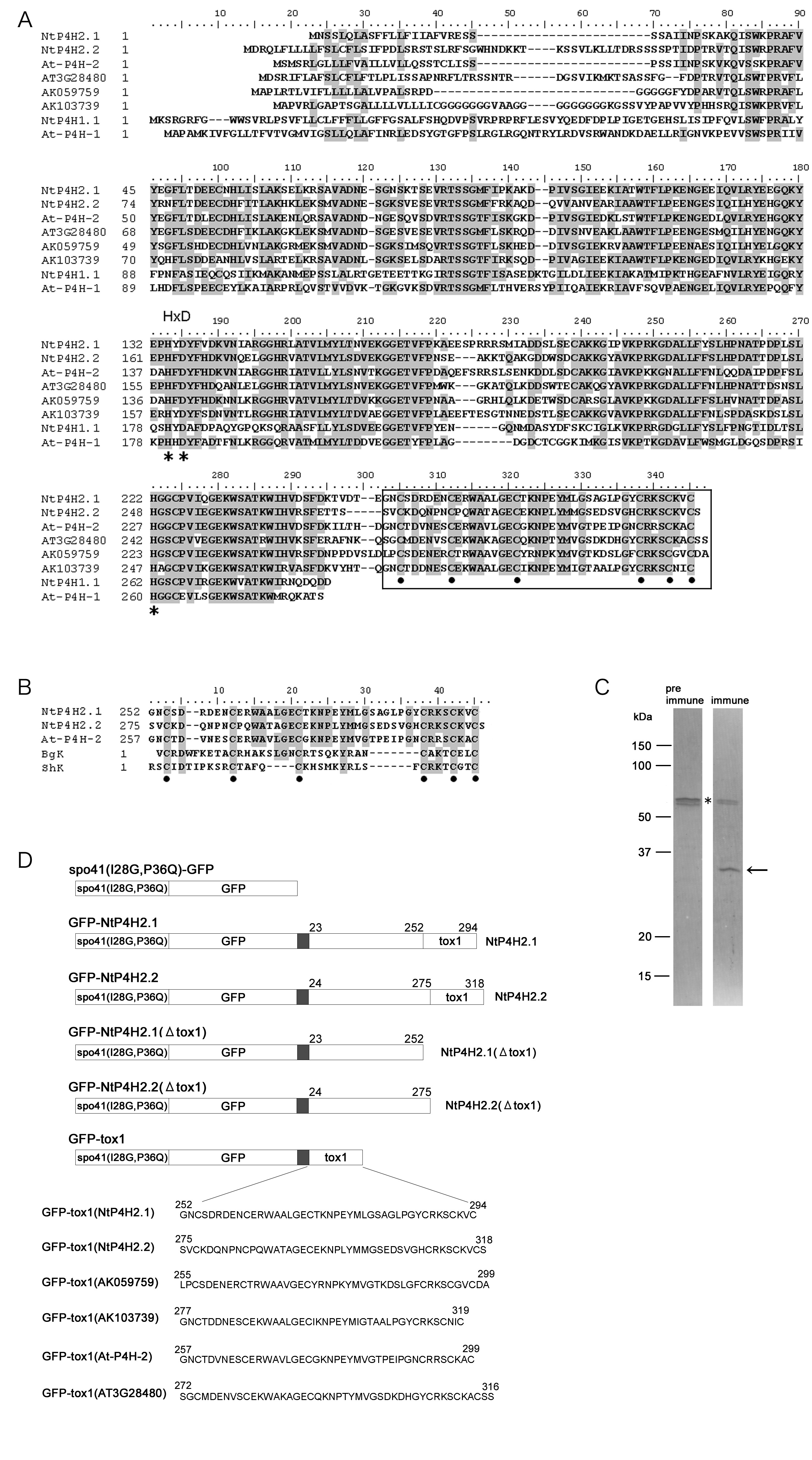

Supplement: Supplementary file 1 [file cells-13-01170-s001.zip › Moriguchi and Matsuoka 3060748 FigureS1.jpg]

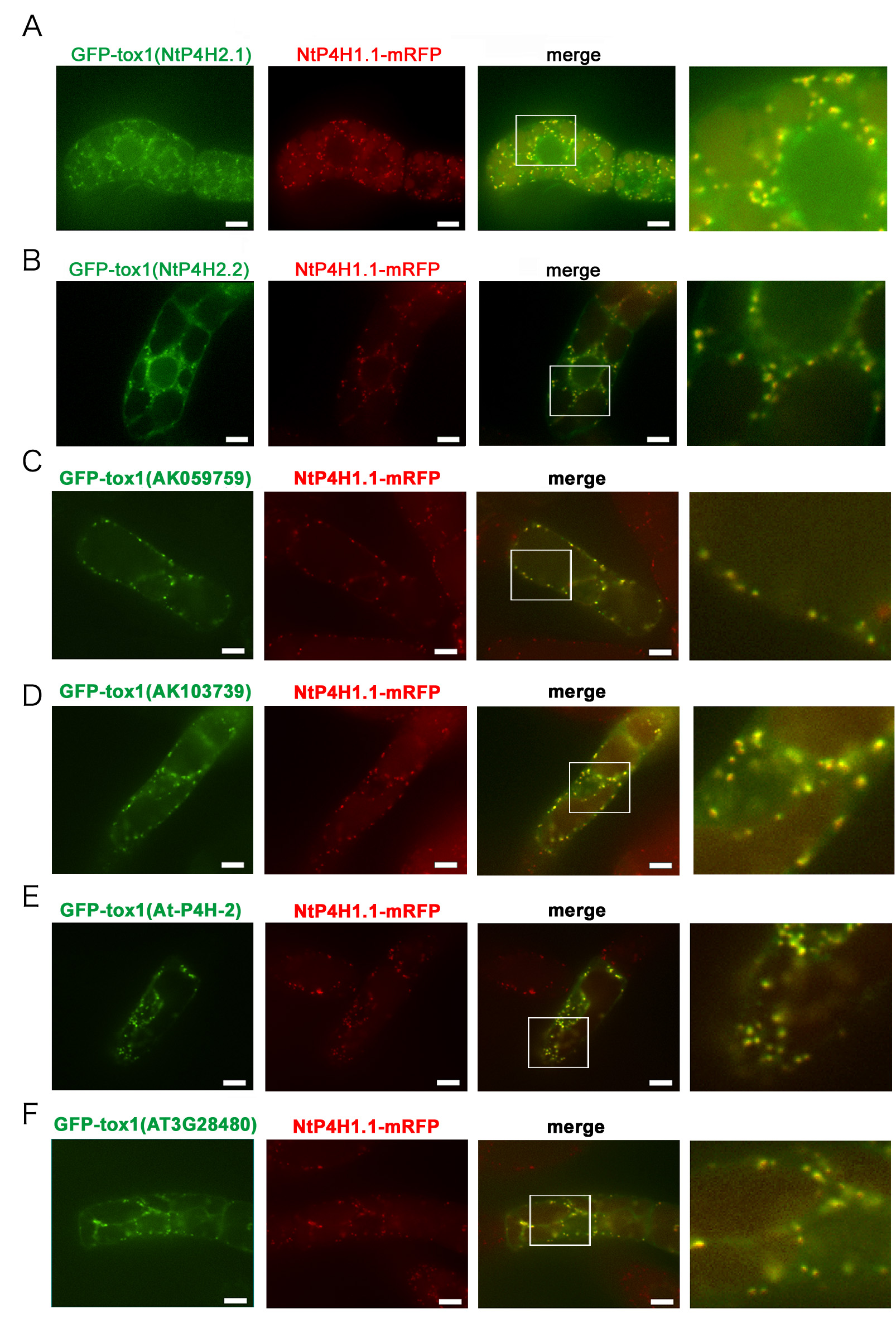

Supplement: Supplementary file 1 [file cells-13-01170-s001.zip › Moriguchi and Matsuoka 3060748 FigureS2.jpg]

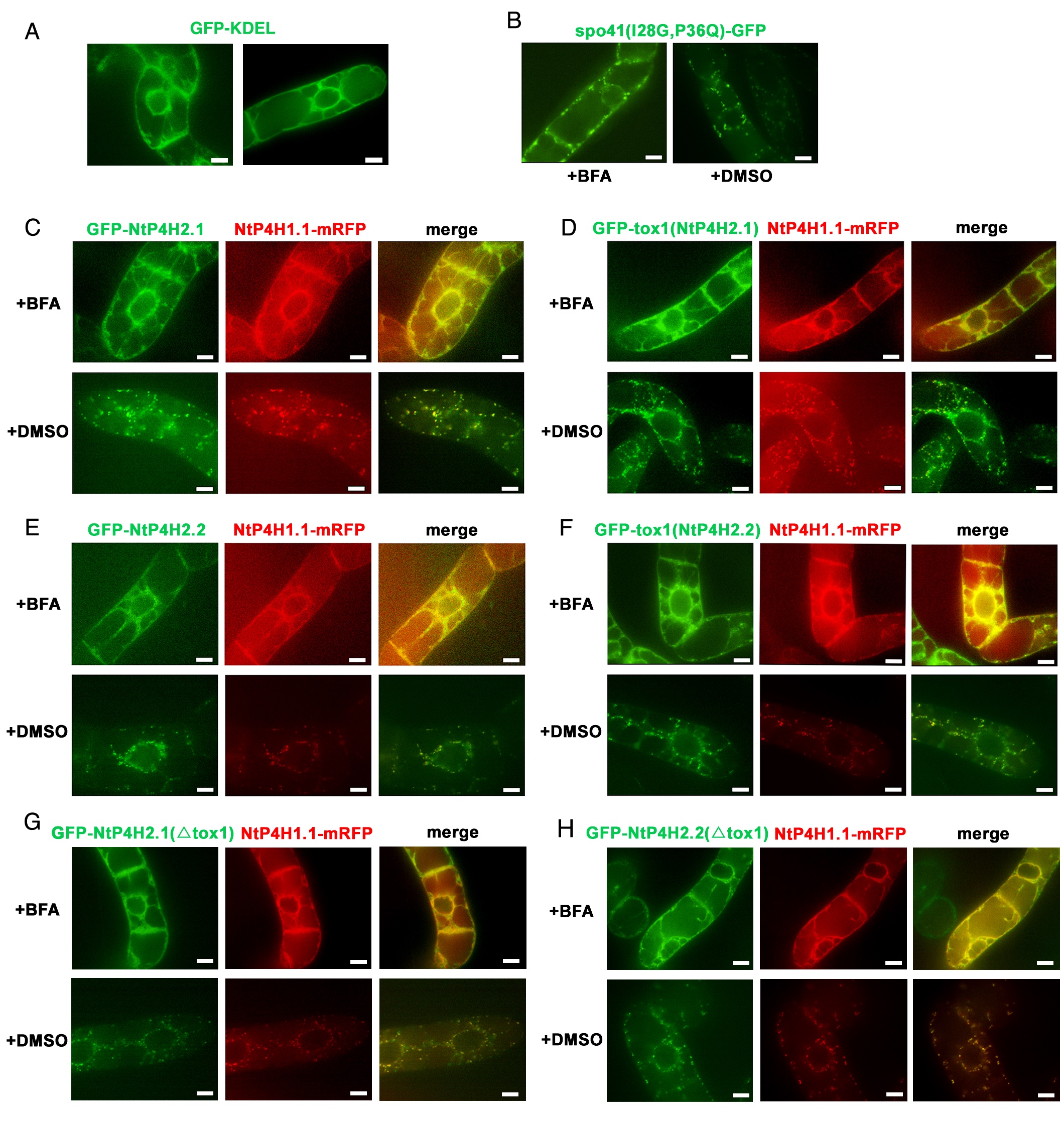

Supplement: Supplementary file 1 [file cells-13-01170-s001.zip › Moriguchi and Matsuoka 3060748 FigureS3.jpg]
